# A134 ENDOSCOPIC RESECTION IS EFFECTIVE FOR COMPLEX LARGE NON-PEDUNCULATED COLORECTAL POLYPS

**DOI:** 10.1093/jcag/gwad061.134

**Published:** 2024-02-14

**Authors:** S X Jiang, A Zarrin, A Walia, C Galorport, W Xiong, R Enns, E Lam, N Shahidi

**Affiliations:** The University of British Columbia Faculty of Medicine, Vancouver, BC, Canada; The University of British Columbia Faculty of Medicine, Vancouver, BC, Canada; St. Paul’s Hospital, Division of Gastroenterology, University of British Columbia, Vancouver, BC, Canada; St. Paul’s Hospital, Division of Gastroenterology, University of British Columbia, Vancouver, BC, Canada; University of British Columbia, Department of Pathology and Laboratory Medicine, Vancouver, BC, Canada; St. Paul’s Hospital, Division of Gastroenterology, University of British Columbia, Vancouver, BC, Canada; St. Paul’s Hospital, Division of Gastroenterology, University of British Columbia, Vancouver, BC, Canada; St. Paul’s Hospital, Division of Gastroenterology, University of British Columbia, Vancouver, BC, Canada

## Abstract

**Background:**

Endoscopic resection is preferred for most large (≥20 mm) non-pedunculated colorectal polyps (LNPCPs) due to superior efficacy, safety, and cost-efficiency compared to surgery. However, endoscopic resection can be challenging due to anatomical location, lesion characteristics, and concomitant colonic disease; these complex LNPCPs (C-LNPCPs) historically have suboptimal outcomes compared to uncomplicated LNPCPs (UC-LNPCPs).

**Aims:**

To compare the outcomes of C-LNPCPs to UC-LNPCPs following endoscopic resection.

**Methods:**

Consecutive patients ampersand:003E 18 years of age who underwent endoscopic resection of a LNPCP were enrolled in a prospective single center observation cohort study (clinicaltrials.gov ID: NCT05402696). C-LNPCP were defined by at least one of the following criteria: involvement of the appendiceal orifice, ileocecal valve, or anorectal junction; previous resection attempt; ≥ 90% circumferential involvement; concomitant inflammatory bowel disease. All other lesions were classified as UC-LNPCPs. Performance was evaluated by technical success (all neoplastic tissue removed at index procedure), procedure-related adverse events (intra-procedural perforation, clinically significant post-endoscopic resection bleeding (CSPEB), delayed perforation, serositis), referral to surgery, and recurrence at first surveillance colonoscopy (SC1).

**Results:**

From 06/2022-09/2023, 350 LNPCPs were referred for endoscopic resection and 335 were attempted (91 C-LNPCPs, 244 UC-LNPCPs). Median age was 68 years (IQR 62-74 years) and 144 (54.1%) were male. Median size was 30mm (IQR 20-40mm). Most lesions were adenomatous (63.7% C-LNPCPs, 64.8% UC-LNPCPs), with cancer identified in 3.3% and 4.9% of C-LNPCPs and UC-LNPCPs, respectively. EMR was the most common resection modality (62.6% C-LNPCPs vs. 64.8% UC-LNPCPs) with C-LNPCPs undergoing more ESD (17.6% vs. 7.0%) and less CSR (19.8% vs. 28.3%; p=0.011). Auxiliary modalities were more frequently used in C-LNPCPs (6.6% vs. 1.2% UC-LNPCPs; p=0.004). Technical success was 95.6% in C-LNPCPs compared to 99.2% in UC-LNPCPs (p=0.049). There was no significant difference between groups in the frequencies of adverse events. 17 LNPCPs (5.1%; 4 C-LNPCP, 13 UC-LNPCPs) underwent surgery (p=1.000). Of those LNPCPs assessed at SC1, there was 1 recurrence (1.0%) in the UC-LNPCP subgroup.

**Conclusions:**

With site-specific technical modifications and mitigating strategies for procedural-related adverse events, endoscopic resection outcomes for C-LNPCPs are comparable to UC-LNPCPs.

**Funding Agencies:**

None

